# The serial mediating effects of different types of academic stress on depression among high school students: a two-layer protective mechanism based on conservation of resources theory

**DOI:** 10.3389/fpsyg.2026.1841587

**Published:** 2026-06-30

**Authors:** Yixuan Duan

**Affiliations:** College of Teacher Education, Ningxia University, Yinchuan, China

**Keywords:** academic stress, depression, loneliness, parent-child communication, perceived stress, self-efficacy

## Abstract

**Objective:**

To examine theoretically informed association patterns linking different types of academic stress to depression among high school students and to investigate the roles of parent–child communication and self-efficacy within a proposed moderated serial mediation model.

**Methods:**

Using convenience sampling, a cross-sectional survey was conducted among 967 high school students in northwestern China. Measures included four types of academic stress (parental, self-imposed, teacher, and social stress), perceived stress (PSS-14), loneliness (UCLA-LS3), parent–child communication (PACS), self-efficacy (GSES), and depression (KADS-11). A moderated serial mediation model was tested with PROCESS Model 83 and 5,000 bootstrap resamples, specifying perceived stress and loneliness as sequential mediators and parent–child communication and self-efficacy as moderators. An EBICglasso psychometric network analysis was further used to provide supplementary descriptive evidence to support the findings.

**Results:**

All four types of academic stress were indirectly associated with depression through the serial pathway involving perceived stress and loneliness, with all 95% confidence intervals excluding zero. Self-imposed stress showed the strongest indirect effect (*b* = 0.036), whereas teacher stress showed the weakest (*b* = 0.013). Social stress additionally showed a distinctive association with loneliness beyond perceived stress (*β* = 0.22, *p* < 0.001), and this indirect effect (*b* = 0.176) was the largest among all pathways. Parent–child communication significantly attenuated the association between self-imposed stress and perceived stress (*β* = −0.19, *p* < 0.001). Self-efficacy significantly attenuated the association between perceived stress and loneliness (*β* = −0.22, *p* < 0.001), showing a stronger moderating association than parent–child communication. In the network analysis, perceived stress showed the highest strength and closeness centrality, self-efficacy showed the highest betweenness centrality, and social stress retained a direct edge with loneliness.

**Conclusion:**

The findings suggest that different types of academic stress may be linked to adolescent depression through a cognitive–interpersonal pathway involving perceived stress and loneliness. Parent–child communication and self-efficacy may serve as protective factors within this association pattern. These results provide a basis for more targeted interventions for adolescent depression in school contexts.

## 1 Introduction

Adolescent depression has become a major concern in both public health and developmental psychology, with the high school period warranting particular attention (Lerner and Steinberg, 2009). On the one hand, adolescents at this stage are confronted with multiple developmental tasks, including pubertal changes, identity integration, and social adaptation. On the other hand, external demands such as school evaluation, academic competition, and expectations regarding future development intensify substantially, rendering high school students a high-risk group for internalizing problems such as depression. National survey data have shown that depressive risk is highly prevalent among Chinese adolescents and increases across grade levels (Tang et al., 2019). More importantly, depression during adolescence is not merely a transient emotional fluctuation; its adverse consequences may extend into adulthood and significantly elevate the risk of severe maladaptive outcomes, including suicidal behavior (Chang et al., 2024). Accordingly, clarifying association patterns related to depression among high school students is not only critical for understanding adolescent mental health problems, but also constitutes an essential prerequisite for early identification and targeted intervention.

Academic stress is not a unitary construct. Xu et al. (2010) classified it into four dimensions—parental stress, self-imposed stress, teacher-related stress, and social stress—corresponding to different ecological layers, namely family, individual, teacher–student, and peer contexts. The first three forms of stress are centered primarily on achievement expectations and competence evaluation, whereas social stress is more directly related to peer comparison and threats to belongingness. Previous research found that excessive parental interference, distant teacher–student relationships, and poor peer relationships were all associated with elevated depressive risk (Tang et al., 2020), suggesting that different sources of stress may be associated with depression through distinct pathways. However, most previous studies have treated academic stress as a single composite variable, paying insufficient attention to the differentiated association pathways of its subtypes. As a result, it remains unclear how stress from different sources is associated with depression and through what pathways such associations may be reflected.

From the perspective of the stress process, academic demands do not automatically translate into depression; rather, individuals’ subjective appraisals constitute a critical intermediary mechanism. Cognitive appraisal theory posits that when individuals evaluate external demands as exceeding their coping resources, perceived stress emerges and subsequently gives rise to psychological distress (Lazarus and Folkman, 1984). A longitudinal study of high school students by Kristensen et al. (2023) further showed that the transmission process linking academic stress to psychological distress was moderated by changes in individuals’ internal resources, thereby underscoring the pivotal role of perceived stress. Nevertheless, perceived stress alone does not provide a sufficient explanation for this process. Depression is associated not only with high-pressure experiences, but also with impaired social connectedness. Loneliness is a subjective negative experience arising from unmet belongingness needs (Baumeister and Leary, 1995). Persistent perceived stress may be associated with lower social connectedness and higher loneliness, which in turn may be linked to depressive symptoms; such associations may be further intensified by rumination and other negative cognitive processes (Nolen-Hoeksema and Watkins, 2011). Meta-analytic evidence has consistently supported the robust association between loneliness and depression (Chen et al., 2023; Mann et al., 2022). Taken together, perceived stress and loneliness may jointly represent a serial association pattern linking cognitive appraisal with interpersonal connectedness. Importantly, because social stress is closely related to interpersonal belonging, it may show an additional association with loneliness beyond perceived stress, thereby showing an association pattern that may be distinct from that of the other three forms of academic stress. This possibility, however, has yet to be tested empirically.

Within this proposed serial association pattern, protective resources may buffer risk at different nodes. Conservation of Resources theory (Hobfoll, 1989) proposes that resource possession serves as an important foundation for coping with stress, and that external family resources and internal personal resources may form a progressive protective structure along the sequence of stress processing. At the family level, parent–child communication constitutes one of the most directly perceived relational resources. Existing review evidence has demonstrated a stable negative association between the quality of parent–child communication and adolescents’ internalizing problems (Zapf et al., 2024). Attachment theory likewise suggests that secure attachment facilitates more adaptive initial appraisals of stressful situations (Bowlby, 1982), thereby buffering the transformation of self-imposed stress into perceived stress. At the individual level, self-efficacy refers to individuals’ generalized belief in their capacity to cope effectively with challenges (Bandura, 1997) and is negatively associated with adolescent depression. Once high perceived stress has emerged, self-efficacy may further determine whether adolescents withdraw relationally: those with high self-efficacy are more likely to adopt active coping strategies and maintain social connectedness, whereas those with low self-efficacy are more likely to retreat into isolation under stress. Self-efficacy may therefore be particularly relevant to the association between perceived stress and loneliness.

Taken together, the existing literature has several limitations. First, the differential association pathways of the four types of academic stress have not been systematically compared within a unified model. Second, although perceived stress and loneliness are both significantly associated with depression, direct evidence remains lacking for integrating them into a serial mediation process from cognitive overload to relational isolation. Third, the node-specific protective effects of parent–child communication and self-efficacy, as well as their relative efficacy, have not been systematically tested. Finally, prior research has relied mainly on variable-centered approaches, with little data-driven network evidence to independently support the proposed pathway structure. To address these gaps, the present study draws on an integrated framework combining cognitive appraisal theory (Lazarus and Folkman, 1984), belongingness theory (Baumeister and Leary, 1995), and Conservation of Resources theory (Hobfoll, 1989), and tests a moderated serial mediation model in a sample of 967 high school students. Specifically, the four types of academic stress are modeled as predictors, perceived stress and loneliness as serial mediators, and depression as the outcome. Parent–child communication is specified as a moderator of the a₁ path, and self-efficacy as a moderator of the a₂ path. Based on this framework, four hypotheses are proposed: H1, all four types of academic stress predict depression through the serial pathway of perceived stress and loneliness, with differences in transmission efficiency across stress types; H2, parent–child communication negatively moderates the association between self-imposed stress and perceived stress; H3, self-efficacy negatively moderates the a₂ path; and H4, self-efficacy will show a stronger moderating association within the proposed serial pathway than parent–child communication.

## 2 Methods

### 2.1 Participants

A cross-sectional questionnaire survey was conducted using convenience sampling in several regular high schools in northwestern China, with first- and second-year high school students as the target participants. The initial sample comprised 1,011 questionnaires. After excluding invalid responses, including patterned responding (e.g., selecting the same option for more than 10 consecutive items), completion times shorter than 3 min, and missing data on key items, 967 valid questionnaires were retained for analysis, yielding a valid response rate of 95.6%. The mean age of the participants was 16.70 years (SD = 0.67). Given the nested structure of the data, intraclass correlation coefficients (ICCs) were calculated for the primary outcome variable (KADS-11) at the classroom and school levels, yielding values of 0.031 and 0.028, respectively — both below the conventional threshold of 0.05 (Hox et al., 2017), indicating negligible clustering effects. Multilevel modeling was therefore not adopted. Sensitivity analyses using cluster-robust standard errors at the school level produced substantively identical results, supporting the robustness of the individual-level analyses. This study was approved by the Academic Ethics Committee of Ningxia University (Approval No. NXU-2025-298). Written informed consent was obtained from all participants prior to data collection, with parental or guardian consent provided for minors (Table 1).

**Table 1:** Demographic characteristics of participants.

Variable	Category	*n*	*%*
Sex	Male	490	50.7%
	Female	477	49.3%
Grade	Grade 10 (Year 1 of senior high)	439	45.4%
	Grade 11 (Year 2 of senior high)	528	54.6%
Ethnicity	Han Chinese	909	94.0%
	Ethnic minority	58	6.0%
Area of residence	Urban	433	44.8%
	Rural	534	55.2%
Only-child status	Only child	271	28.0%
	Non-only child	696	72.0%
Family structure	Single-parent household	87	9.0%
	Two-parent household	880	91.0%
Class officer	Yes	435	45.0%
	No	532	55.0%
Boarding status	Boarding student	532	55.0%
	Non-boarding student	435	45.0%

### 2.2 Measures

#### 2.2.1 Academic stress

Academic stress was assessed using the Academic Stress Questionnaire for Middle School Students (Xu et al., 2010), which has been validated in Chinese high school samples (Ma et al., 2025). The 21-item questionnaire comprises four subscales: parental stress (6 items), self-imposed stress (6 items), teacher stress (5 items), and social stress (4 items). Items are rated on a 5-point Likert scale (1 = strongly disagree to 5 = strongly agree), with higher scores indicating greater stress on each subscale. The four-factor structure was originally established through exploratory factor analysis and was further supported in subsequent Chinese adolescent samples (Ma et al., 2025), providing a sound structural basis for composite score use. The total scale demonstrated satisfactory internal consistency (Cronbach’s *α* = 0.81, McDonald’s *ω* = 0.83). At the subscale level, Cronbach’s α coefficients were 0.76, 0.73, 0.63, and 0.63 for parental, self-imposed, teacher, and social stress, respectively; supplementary McDonald’s ω coefficients were 0.78, 0.75, 0.74, and 0.73, all exceeding the conventional threshold of 0.70. The relatively lower *α* values for the teacher and social stress subscales are partly attributable to their shorter length (5 and 4 items, respectively), and the ω coefficients confirmed adequate reliability for all subscales.

#### 2.2.2 Perceived stress

Perceived stress was measured with the 14-item Perceived Stress Scale (PSS-14; Cohen et al., 1983), as adapted for Chinese populations by Yang and Huang (2003). Items are rated on a 5-point frequency scale (0 = never to 4 = always), with total scores ranging from 0 to 56; higher scores indicate greater perceived stress. Cronbach’s *α* = 0.88.

#### 2.2.3 Loneliness

Loneliness was assessed with the UCLA Loneliness Scale, Version 3 (UCLA-LS3; Russell, 1996), validated for use with Chinese adolescents by Ip et al. (2024). The 20-item scale uses a 4-point response format (1 = never to 4 = always), with total scores ranging from 20 to 80; higher scores indicate greater loneliness. Cronbach’s *α* = 0.83.

#### 2.2.4 Parent–child communication

Parent–child communication quality was assessed with the Parent-Adolescent Communication Scale (PACS; Barnes and Olson, 1982), which has been used in Chinese adolescent samples (Xia et al., 2004). The scale comprises two 10-item subscales: open communication and problematic communication, the latter reverse-scored. Items are rated on a 5-point Likert scale, with total scores ranging from 20 to 100; higher scores reflect better communication quality. Cronbach’s *α* = 0.84.

#### 2.2.5 Self-efficacy

General self-efficacy was measured with the General Self-Efficacy Scale (GSES; Schwarzer and Jerusalem, 1995), adapted for Chinese populations by Wang et al. (2001). The 10-item scale uses a 4-point response format (1 = not at all true to 4 = exactly true), with total scores ranging from 10 to 40; higher scores indicate stronger self-efficacy beliefs. Cronbach’s *α* = 0.82.

#### 2.2.6 Depression

Depressive symptoms were assessed with the 11-item Kutcher Adolescent Depression Scale (KADS-11; Brooks et al., 2003), the Chinese version of which has been validated in Chinese adolescent samples by Zhou et al. (2016). Each item reflects a core symptom of depression (e.g., low mood, sleep difficulties, decreased interest in activities), rated on a 4-point frequency scale (0 = hardly ever to 3 = all of the time) based on experiences over the past week; higher scores indicate more severe depressive symptoms. Cronbach’s α = 0.87.

### 2.3 Common method Bias

Procedural controls were implemented to minimize common method bias, including anonymous responding, randomized scale ordering, and the inclusion of reverse-scored items. A Harman’s single-factor test was conducted as a *post hoc* statistical check. Exploratory factor analysis yielded nine factors with eigenvalues greater than 1, with the first factor accounting for 19.83% of the total variance — well below the 40% threshold recommended by Podsakoff et al. (2003). These results suggest that common method bias did not pose a substantial threat to the validity of the findings.

### 2.4 Data analysis

Descriptive statistics and Pearson correlations were computed using SPSS 26.0. The moderated serial mediation model was estimated using the PROCESS macro (Model 83; Hayes, 2018). This regression-based approach used observed composite scores rather than latent variables and therefore did not explicitly model measurement error. The model specified four dimensions of academic stress as predictors (X₁ = parental stress, X₂ = self-imposed stress, X₃ = teacher stress, X₄ = social stress), perceived stress (M₁) and loneliness (M₂) as sequential mediators, and depression (Y) as the outcome variable. Parent–child communication (W₁) was specified as a moderator of the path from self-imposed stress to perceived stress, and self-efficacy (W₂) was specified as a moderator of the path from perceived stress to loneliness. All continuous variables were mean-centered prior to the computation of interaction terms (Aiken and West, 1991). Indirect effects were estimated using bias-corrected bootstrap confidence intervals (CIs) based on 5,000 resamples; an effect was considered statistically significant if the 95% CI excluded zero. The index of moderated mediation was likewise evaluated using bootstrap 95% CIs (Hayes, 2018).

## 3 Results

### 3.1 Descriptive statistics and correlational analysis

Descriptive statistics and the intercorrelation matrix for all study variables are presented in Table 2 (*N* = 967). Among the four dimensions of academic stress, self-imposed stress had the highest mean (*M* = 22.11, *SD* = 4.69), followed by parental stress (*M* = 19.19, *SD* = 5.06), teacher stress (*M* = 17.92, *SD* = 4.82), and social stress (*M* = 11.14, *SD* = 1.97); the mean for self-imposed stress fell in the upper-moderate range of its scale, suggesting a notable degree of internalized achievement expectations. Perceived stress was at a moderate level (*M* = 24.56, *SD* = 7.29), loneliness was slightly above the midpoint of its 20–80 scale (*M* = 39.09, *SD* = 9.09), and depression had a mean of 5.90 (*SD* = 3.86).

**Table 2:** Descriptive statistics, internal consistency coefficients, and pearson correlations among study variables.

Variable	M	SD	1	2	3	4	5	6	7	8	9
Parental stress	19.19	5.06	—								
Self-imposed stress	22.11	4.69	0.46^***^	—							
Teacher stress	17.92	4.82	0.38^***^	0.45^***^	—						
Social stress	11.14	1.97	0.26^***^	0.31^***^	0.27^***^	—					
Perceived stress	24.56	7.29	0.59^***^	0.61^***^	0.47^***^	0.38^***^	—				
Loneliness	39.09	9.09	0.33^***^	0.39^***^	0.32^***^	0.39^***^	0.52^***^	—			
Depression	5.90	3.86	0.40^***^	0.41^***^	0.34^***^	0.32^***^	0.54^***^	0.62^***^	—		
Parent–child communication	41.12	8.13	−0.32^***^	−0.31^***^	−0.25^***^	−0.14^***^	−0.31^***^	−0.24^***^	−0.43^***^	—	
Self-efficacy	27.34	6.41	−0.26^***^	−0.34^***^	−0.20^***^	−0.12^***^	−0.26^***^	−0.24***	−0.44^***^	0.38^***^	—

^***^p < 0.001.

Bivariate correlations indicated that all four dimensions of academic stress were significantly and positively associated with perceived stress (*r*s = 0.38–0.61), loneliness (*r*s = 0.32–0.39), and depression (*r*s = 0.32–0.41; all *p*s < 0.001). Self-imposed stress showed the strongest correlation with perceived stress (*r* = 0.61), followed by parental stress (*r* = 0.59); both exceeded the correlations for teacher stress (*r* = 0.47) and social stress (*r* = 0.38). Social stress showed the highest correlation with loneliness among the four stress dimensions (*r* = 0.39); however, a Fisher *z*-transformation test indicated that this difference relative to the teacher stress–loneliness correlation did not reach statistical significance (*z* = 1.75, *p* = 0.080). Both protective resources were significantly and negatively correlated with all mediators and the outcome variable (all *p*s < 0.001).

### 3.2 Moderated serial mediation analysis

Using the four types of academic stress as predictors, perceived stress (M₁) and loneliness (M₂) as sequential mediators, and depression (Y) as the outcome variable, a moderated serial mediation analysis was conducted with PROCESS Model 83. The results of the three regression equations are presented in Table 3.

**Table 3:** Results of moderated serial mediation analysis.

Predictor	*B*	*SE*	*β*	*t*	*p*
Outcome: Perceived stress (M₁) *R*^2^ = 0.58, F(9, 957) = 145.72, *p* < 0.001					
Parental stress (X₁)	0.48	0.04	0.33	13.27	< 0.001
Self-imposed stress (X₂)	0.54	0.04	0.35	13.37	< 0.001
Teacher stress (X₃)	0.20	0.04	0.13	5.34	< 0.001
Social stress (X₄)	0.48	0.08	0.13	5.78	< 0.001
Parent–child communication (W₁)	−0.03	0.02	−0.04	−1.62	0.106
W₁ × Self-imposed stress	−0.03	0.005	−0.19	−7.21	< 0.001
Outcome: Loneliness (M₂) *R*^2^ = 0.37, F(9, 957) = 63.01, *p* < 0.001					
Perceived stress (M₁)	0.39	0.05	0.31	8.20	< 0.001
Social stress (X₄, extra direct effect)	1.03	0.13	0.22	7.93	< 0.001
Self-efficacy (W₂)	−0.12	0.04	−0.08	−2.97	0.003
W₂ × Perceived stress	−0.04	0.005	−0.22	−8.51	< 0.001
Outcome: Depression (Y) *R*^2^ = 0.55, F(10, 956) = 116.66, *p* < 0.001					
Loneliness (M₂, path b)	0.17	0.01	0.40	15.26	< 0.001
Perceived stress (M₁, direct effect c′)	0.11	0.02	0.20	6.00	< 0.001
Parent–child communication (W₁)	−0.09	0.01	−0.18	−7.44	< 0.001
Self-efficacy (W₂)	−0.13	0.01	−0.22	−8.99	< 0.001

B, unstandardized coefficient; β, standardized coefficient. R^2^ and F statistics are reported in each subgroup heading. Covariates (gender, urban/rural) are included but not shown. All continuous predictors were mean-centered prior to computing interaction terms. The a₁-path interaction term is represented by W₁ × Self-imposed stress.

In Equation 1, with perceived stress as the dependent variable, the model was significant, R^2^ = 0.58, *F*(9, 957) = 145.72, *p* < 0.001. All four types of academic stress significantly and positively predicted perceived stress. Specifically, the predictive effects of parental stress (*β* = 0.33) and self-imposed stress (*β* = 0.35) were both greater than those of teacher stress (*β* = 0.13) and social stress (*β* = 0.13), and the 95% confidence intervals from the Bootstrap difference tests did not include zero. The main effect of parent–child communication was not significant (*β* = −0.04, *p* = 0.106), whereas the interaction between parent–child communication and self-imposed stress significantly and negatively predicted perceived stress (*β* = −0.19, *p* < 0.001), supporting H₂. Thus, the moderating role of parent–child communication should be interpreted as specific to the association between self-imposed stress and perceived stress, rather than as a general moderating effect across all dimensions of academic stress. The simple slope analysis further indicated that the predictive effect of self-imposed stress on perceived stress was significant at all levels of parent–child communication (*ps* < 0.001) and decreased as the quality of parent–child communication increased; moreover, the Bootstrap 95% confidence interval for the difference in slopes between the high- and low-communication groups did not include zero ([0.40, 0.72]).

In Equation 2, with loneliness as the dependent variable, the model was significant, *R*^2^ = 0.37, *F*(9, 957) = 63.01, *p* < 0.001. Perceived stress significantly and positively predicted loneliness (*β* = 0.31, *p* < 0.001). After controlling for perceived stress, social stress still showed a significant positive predictive effect on loneliness (β = 0.22, *p* < 0.001), whereas the predictive effects of parental stress (*p* = 0.992), self-imposed stress (*p* = 0.085), and teacher stress (*p* = 0.085) were not significant. In addition, the interaction between self-efficacy and perceived stress significantly and negatively predicted loneliness (*β* = −0.22, *p* < 0.001), supporting H₃. The simple slope analysis showed that the effect of perceived stress on loneliness remained positive across different levels of self-efficacy, with a stronger effect in the low-self-efficacy group and a weaker, though still significant, effect in the high-self-efficacy group (*p* = 0.038).

In Equation 3, with depression as the dependent variable, the model was significant, *R*^2^ = 0.55, *F*(10, 956) = 116.66, *p* < 0.001. Both loneliness (*β* = 0.40, *p* < 0.001) and perceived stress (*β* = 0.20, *p* < 0.001) significantly and positively predicted depression. In addition, both parent–child communication (*β* = −0.18, *p* < 0.001) and self-efficacy (*β* = −0.22, *p* < 0.001) showed significant negative associations with depression. After the mediating and moderating variables were included in the model, the direct effects of the four types of academic stress on depression were no longer significant (*ps* = 0.17–0.54), suggesting that their associations with depression were primarily reflected through the indirect pathways examined in this study.

### 3.3 Bootstrap tests of indirect effects

The results of the Bootstrap tests for the indirect effects are presented in Table 4. The 95% confidence intervals for all nine indirect pathways did not include zero, indicating that all indirect effects were statistically significant. Among the four simple mediation pathways, the indirect effect of self-imposed stress on depression through perceived stress was the largest (effect = 0.057, 95% *CI* [0.038, 0.078]) and was significantly greater than that of the teacher-related stress pathway (*Δ* = 0.036, 95% *CI* [0.015, 0.060]). Among the four serial mediation pathways, the indirect effect associated with self-imposed stress was also the largest (effect = 0.036, 95% *CI* [0.025, 0.048]). In addition, the indirect effect of social stress on depression through loneliness was the largest among all indirect pathways (effect = 0.176, 95% *CI* [0.125, 0.230]), indicating that loneliness may play a particularly important role in the association between social stress and depression.

**Table 4:** Bootstrap indirect effects and 95% confidence intervals.

Indirect effect pathway	Effect	*Boot SE*	95% CI
Single mediation: Academic stress → Perceived stress → Depression			
Parental stress	0.050	0.009	[0.034, 0.068]
Self-imposed stress	0.057	0.010	[0.038, 0.078]
Teacher stress	0.021	0.005	[0.012, 0.031]
Social stress	0.051	0.012	[0.030, 0.076]
Chain mediation: Academic stress → Perceived stress → Loneliness → Depression			
Parental stress	0.032	0.005	[0.022, 0.042]
Self-imposed stress†	0.036	0.006	[0.025, 0.048]
Teacher stress	0.013	0.003	[0.008, 0.020]
Social stress	0.032	0.007	[0.020, 0.047]
Extra direct path (social stress specific)			
Social stress → Loneliness → Depression‡	0.176	0.027	[0.125, 0.230]

Boot SE = bootstrapped standard error. 95% CI = 95% bootstrapped confidence interval; all reported CIs exclude zero, indicating that all indirect effects are statistically significant. †Largest indirect effect among the four chain-mediated pathways. ‡Largest indirect effect overall across all nine pathways.

### 3.4 Conditional indirect effects at different levels of the moderators

Using self-imposed stress, which showed the largest serial indirect effect, as the focal predictor, the conditional indirect effect through perceived stress and loneliness was estimated at three levels of each moderator (M − 1 SD, M, and M + 1 SD). The results are presented in Table 5.

**Table 5:** Conditional indirect effects of self-imposed stress on depression at three levels of each moderator.

Moderator level	Effect	*Boot SE*	95% CI
Parent–child communication (W₁) moderating the a₁ path			
Low (M − 1SD = 32.99)	0.054	0.009	[0.039, 0.072]
Mean (M = 41.12)	0.036	0.006	[0.025, 0.048]
High (M + 1SD = 49.25)	0.017	0.004	[0.009, 0.027]
Self-efficacy (W₂) moderating the a₂ path			
Low (M − 1SD = 20.94)	0.061	0.008	[0.046, 0.077]
Mean (M = 27.34)	0.036	0.006	[0.025, 0.048]
High (M + 1SD = 33.75)	0.011	0.006	[0.000, 0.022]

The index of moderated mediation for parent–child communication was significant (Index = −0.002, 95% *CI* [−0.003, −0.002]). The serial indirect effect decreased as the level of parent–child communication increased, from 0.054 (95% CI [0.039, 0.072]) at the low level to 0.017 (95% CI [0.009, 0.027]) at the high level. Because the 95% confidence intervals at all three levels excluded zero, the indirect effect remained significant across levels of parent–child communication, although its magnitude was attenuated at higher levels.

The index of moderated mediation for self-efficacy was also significant (Index = −0.004, 95% *CI* [−0.005, −0.003]). The serial indirect effect decreased from 0.061 (95% CI [0.046, 0.077]) at the low level of self-efficacy to 0.011 (95% *CI* [0.000, 0.022]) at the high level. Although the indirect effect was markedly smaller at higher levels of self-efficacy, it should be interpreted cautiously because the lower bound of the confidence interval approached zero.

**Figure 1:**
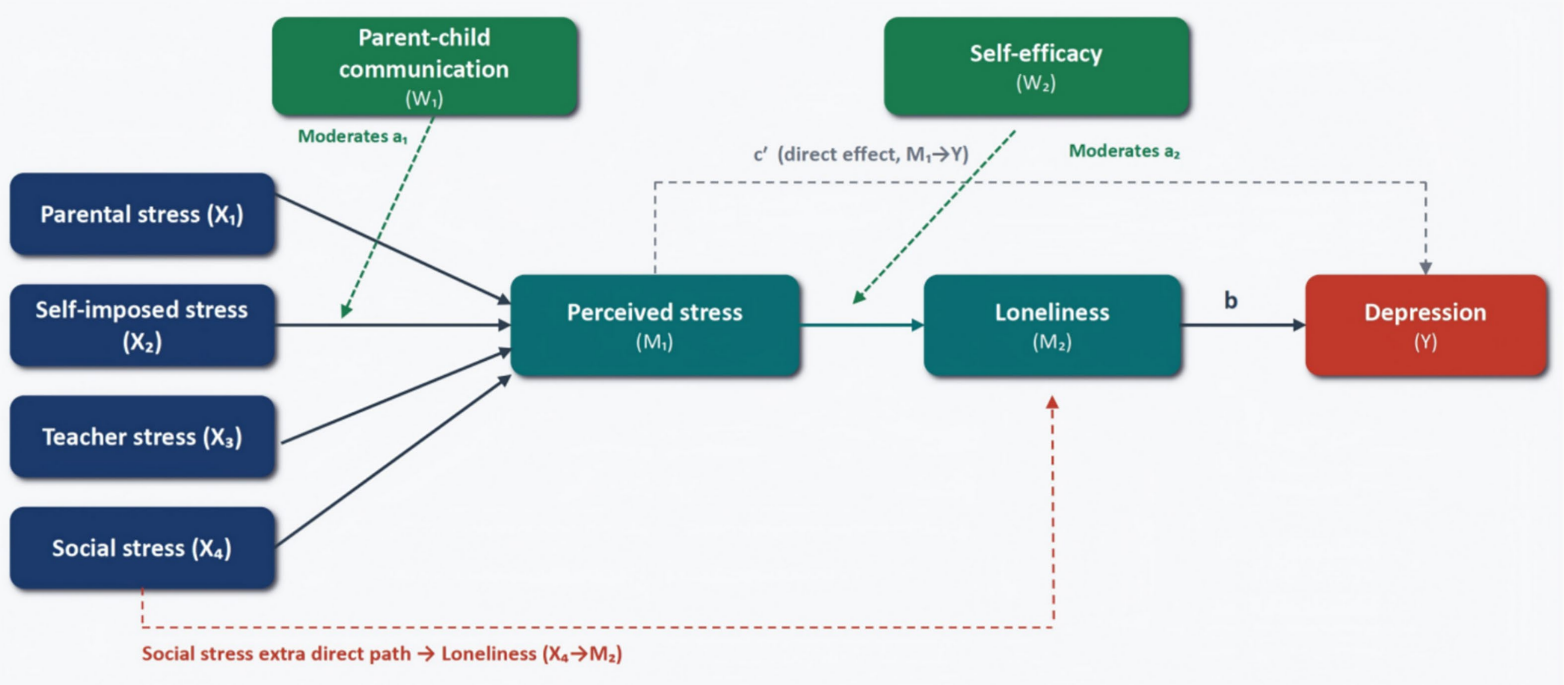
Moderated serial mediation model of academic stress and depression.

A comparison of the two indices of moderated mediation showed that the bootstrap 95% confidence interval for their difference excluded zero (*Δ* = −0.002, 95% *CI* [−0.003, −0.001]), suggesting that self-efficacy showed a stronger moderating association than parent–child communication in the present model. Overall, these results indicate that the two protective factors may be related to different segments of the serial pathway (see Figure 1).

### 3.5 Network analysis results

To complement the regression-based findings, an EBICglasso network (*γ* = 0.50) was estimated for the nine core variables, and edge stability was evaluated using 500 nonparametric bootstrap samples. The final network retained 21 stable edges, with a density of 0.58. Case-dropping bootstrap analyses indicated acceptable stability for the centrality indices.

**Figure 2:**
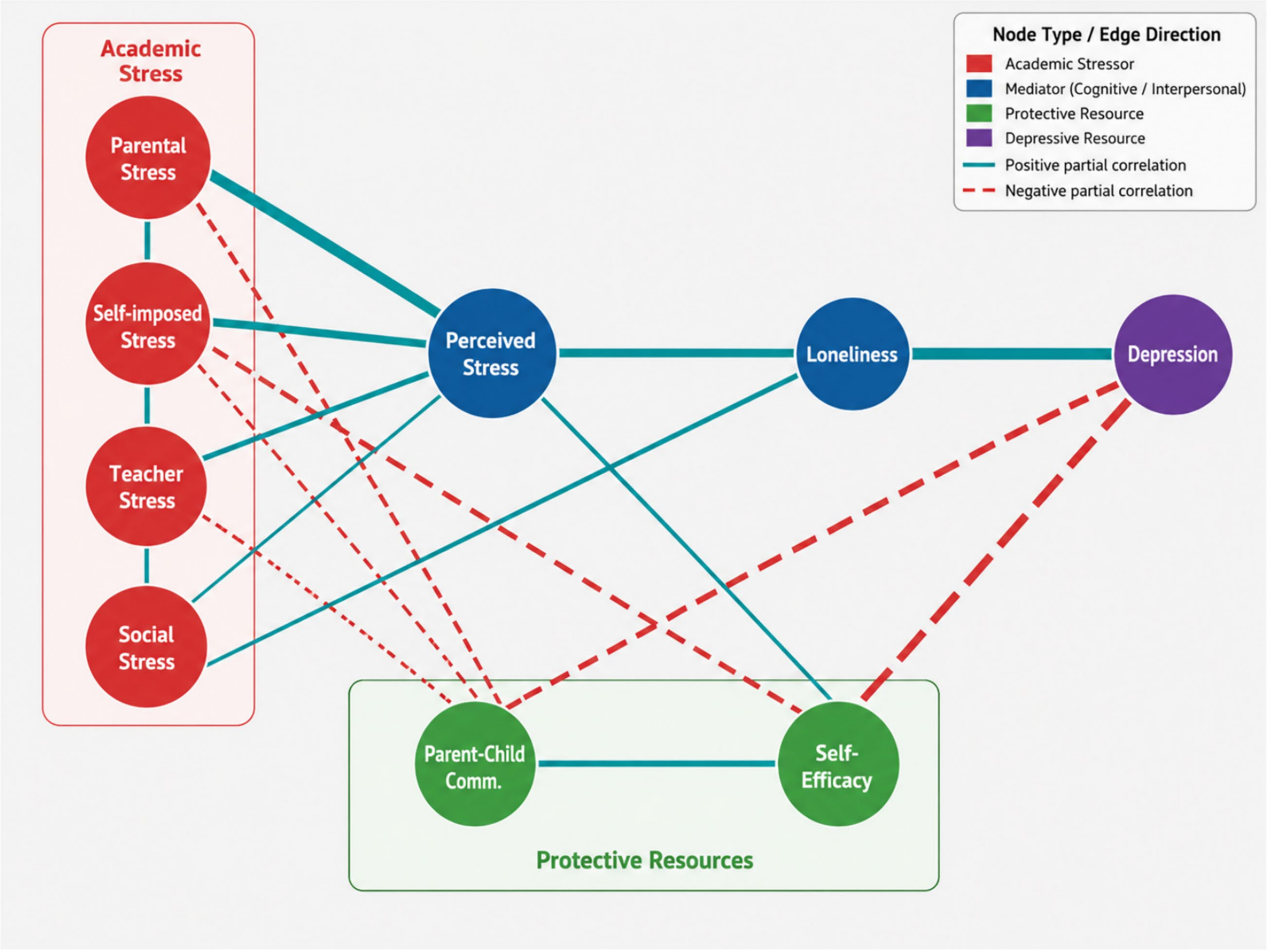
Psychometric network of academic stress and depression among high school students.

**Figure 3:**
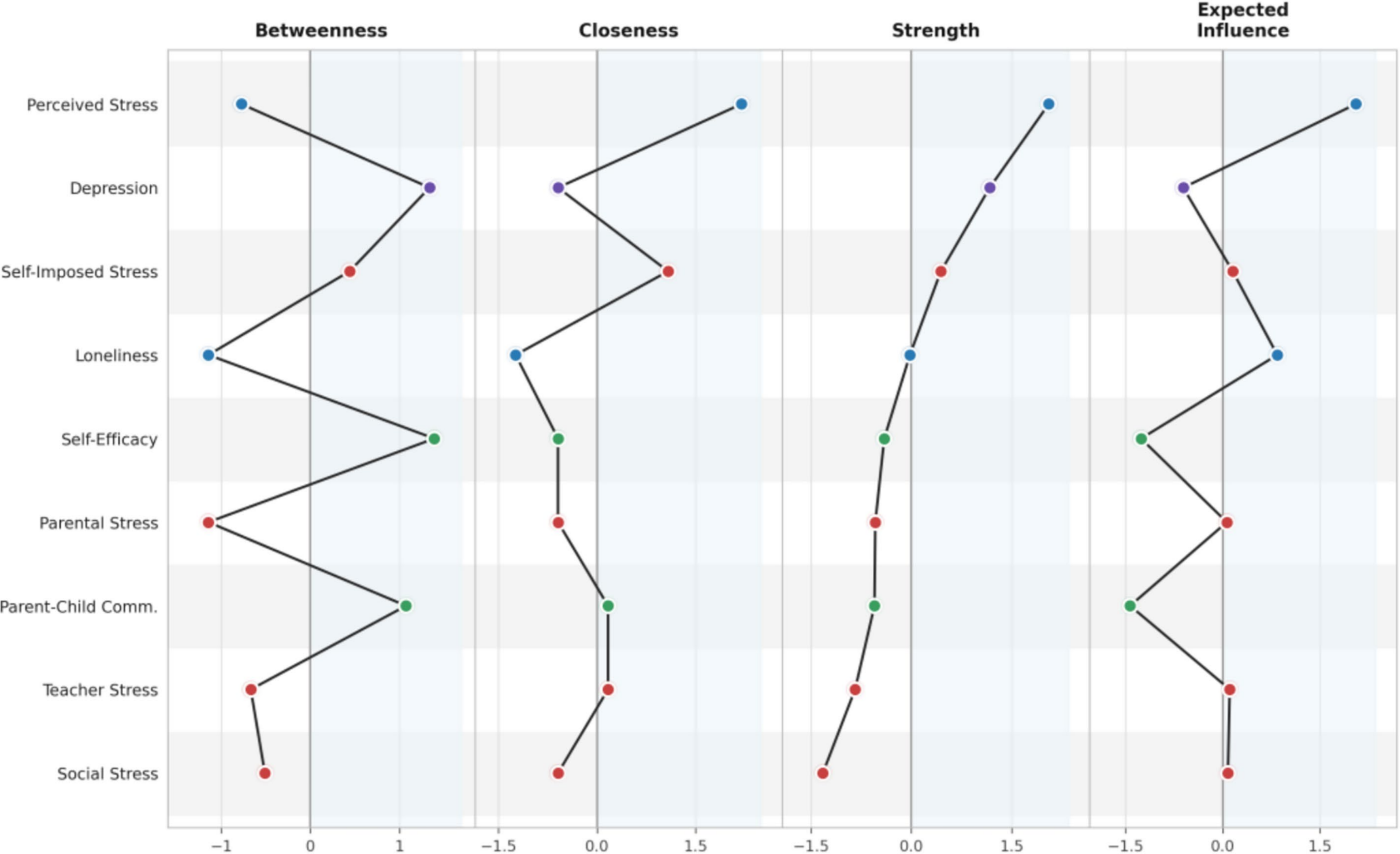
Node centrality indices of the psychometric network.

As shown in Figures 2, 3, perceived stress exhibited the highest strength (1.33) and closeness centrality (0.89), indicating that it occupied a relatively central position in the network. Self-efficacy showed the highest betweenness centrality (0.86), suggesting that it may play an important bridging role in the overall pattern of associations. In terms of edge weights, the loneliness–depression edge (*r* = 0.43, 95% *CI* [0.38, 0.47]) was the strongest, followed by the parental stress–perceived stress edge (*r* = 0.35) and the self-imposed stress–perceived stress edge (*r* = 0.33). Notably, a stable direct edge was observed between social stress and loneliness (*r* = 0.19), whereas such an edge was not observed for the other three stress dimensions. Overall, the network results were broadly consistent with the regression findings, although they should be interpreted as supplementary descriptive evidence rather than confirmatory tests of the proposed mediation model.

## 4 Discussion

Based on a sample of 967 high school students, this study examined the association patterns linking four types of academic stress to depression through a proposed serial pathway involving perceived stress and loneliness, as well as the moderating roles of parent–child communication and self-efficacy within this pathway. The results supported all four hypotheses and further revealed three major findings: first, different types of academic stress differed in the strength of their indirect associations with depression; second, social stress exhibited a distinctive pathway to loneliness independent of perceived stress; and third, parent–child communication and self-efficacy showed differentiated moderating associations at different nodes of the proposed pathway, a pattern consistent with a node-specific two-layer protective structure.

### 4.1 Differential associations between four types of academic stress and perceived stress

The results showed that parental stress and self-imposed stress were the strongest predictors of perceived stress, with comparable effect sizes, and both were significantly stronger than teacher-related stress and social stress. This finding is generally consistent with that of Ma et al. (2025), who also found that stressors originating from adolescents’ core microsystems have stronger situational penetration and personal relevance (Bronfenbrenner, 1986), and are therefore more likely to be subjectively appraised as burdens exceeding one’s coping resources.

Particularly noteworthy is the finding that self-imposed stress had a slightly stronger predictive effect than parental stress. According to cognitive appraisal theory (Lazarus and Folkman, 1984), stress is shaped not merely by objective demands themselves, but by whether individuals appraise such demands as threats exceeding their available resources. Self-imposed stress reflects the discrepancy between internalized achievement standards and actual performance. This tension, which is actively maintained by the individual, is less likely to be alleviated by contextual changes or external reassurance than externally imposed demands. As such, self-imposed stress is more likely to act on the cognitive appraisal system in a persistent, internalized, and self-reinforcing manner, and may therefore show a stronger association with perceived stress. This finding suggests that, in the highly competitive context of the college entrance examination, internalized achievement expectations may have become a major trigger of perceived stress among high school students, a pattern that previous studies treating academic stress as a single composite construct may have failed to detect.

The network analysis corroborated this pattern: perceived stress had the highest node strength and closeness centrality in the network, providing descriptive evidence consistent with its central position in the stress–depression association network.

By contrast, teacher-related stress and social stress showed relatively weaker predictive effects on perceived stress. This may suggest that although stress arising from teacher–student interactions and peer comparisons also has psychological consequences, it may not initially manifest as an obvious sense of subjective loss of control. Instead, it may require additional cognitive or situational processing before gradually being transformed into perceived stress (Kristensen et al., 2023). In other words, although all four forms of academic stress were associated with perceived stress, they differed in the strength of these associations.

### 4.2 Serial mediation pattern and the distinctive association of social stress with loneliness

The present study provides evidence consistent with a serial mediation pattern linking academic stress, perceived stress, loneliness, and depression. Specifically, academic stress was positively associated with perceived stress, which in turn was positively associated with both loneliness and depressive symptoms. This pattern suggests that the association between academic stress and depression may operate through both cognitive appraisal processes and interpersonal pathways involving loneliness, rather than being limited to a direct association. By integrating perceived stress and loneliness into the same model, the present findings extend prior research on academic stress and adolescent depression and are consistent with the view that perceived stress and loneliness may function as two related yet distinguishable mediating variables within the academic stress–depression association. Longitudinal evidence similarly identified loneliness as an important correlate in the association between academic stress and emotional distress among Chinese secondary school students (Wang et al., 2025).

Among the four types of academic stress, self-imposed stress showed the strongest chained effect, indicating that it not only more readily activates perceived stress but also more easily extends subjective loss of control into the interpersonal domain. One possible explanation is that once internalized achievement expectations are frustrated, individuals are more likely to attribute failure to stable and pervasive personal deficiencies rather than to external conditions or specific task difficulty. Such generalized self-devaluation is more likely to be accompanied by shame, withdrawal, and self-isolation, thereby increasing loneliness (Nolen-Hoeksema and Watkins, 2011).

The dual-path structure of social stress is one of the most theoretically meaningful findings of this study. The results showed that, even after controlling for perceived stress, social stress still significantly and positively predicted loneliness, whereas the additional direct effects of the other three types of stress were all non-significant. This social stress was associated with depression not only through the general pathway involving perceived stress and loneliness, but also through an additional pathway involving loneliness after perceived stress was controlled. This pattern suggests that social stress may be linked to adolescents’ sense of belonging in ways that are not fully captured by perceived stress. This finding is consistent with belongingness theory (Baumeister and Leary, 1995): for adolescents, peer acceptance, social comparison, and group inclusion are core developmental concerns. When adolescents experience peer rejection, competitive comparison, or social threat in academic contexts, social stress may be closely related to adolescents’ sense of belonging and loneliness in ways that are not fully accounted for by perceived stress. This interpretation is also consistent with prior evidence suggesting that peer relationship quality is one of the most proximal predictors of adolescent loneliness, and that its association with loneliness may not be fully explained by general stress appraisal processes (Verity et al., 2025).

Furthermore, the effect size of this pathway of social stress was the largest among all indirect effects, suggesting that threats in peer relationships may exert even stronger detrimental effects on loneliness and depression than some stressors traditionally defined as “academic demands.” This finding implies that interventions targeting depression in high school students may still fail to address key risk points if they focus solely on academic burden and general stress management. By contrast, improving peer relationship quality, buffering against competitive social comparison, and enhancing students’ sense of group belonging may represent more targeted intervention directions. The network analysis further corroborated this finding: social stress was the only academic stress node with a direct stable edge to loneliness, while the other three stress types showed no such direct connection, providing supplementary descriptive evidence consistent with its distinctive association with belongingness-related experiences.

### 4.3 Node-specific protective associations of parent–child communication and self-efficacy

This study further found that parent–child communication and self-efficacy showed differentiated moderating associations within the proposed stress–depression pathway. Specifically, parent–child communication significantly moderated the association between self-imposed stress and perceived stress, suggesting that higher levels of parent–child communication were associated with a weaker link between self-imposed stress and perceived stress. One possible explanation is that self-imposed stress reflects adolescents’ internalized achievement expectations and self-regulatory demands, which may be shaped by family expectations and parent–child interactions (Chen et al., 2023; Song et al., 2024). High-quality parent–child communication may help adolescents clarify academic expectations and appraise these demands as more manageable, thereby attenuating the association between self-imposed stress and perceived stress. Secure parent–child relationships may provide adolescents with stable emotional support and a basis for cognitive reappraisal, which could facilitate more adaptive interpretations of academic demands (Bowlby, 1982). This interpretation is also consistent with prior review evidence highlighting the relevance of parent–child communication to adolescents’ internalizing problems (Zapf et al., 2024).

Self-efficacy significantly moderated the association between perceived stress and loneliness. The results showed that the serial indirect association was substantially reduced among adolescents with higher self-efficacy. Although the simple slope of perceived stress predicting loneliness remained significant in the high self-efficacy group, its effect size was very small, suggesting that higher self-efficacy was associated with a substantially weaker link between perceived stress and loneliness. Individuals with stronger efficacy beliefs are more likely to maintain a problem-solving orientation and social engagement when facing stressful situations, whereas those with lower self-efficacy may be more prone to social withdrawal under perceived stress (Bandura, 1997). Longitudinal evidence similarly indicates that self-efficacy may buffer the association between academic stress and psychological distress (Kristensen et al., 2023). The present findings extend this evidence by suggesting that self-efficacy may be especially relevant to the association between perceived stress and loneliness in the proposed pathway.

A comparison of the two moderating effects indicates a meaningful pattern of node-specific differentiation, though its interpretation warrants caution given the cross-sectional design of the study. Parent–child communication showed a stronger moderating association for the link between self-imposed stress and perceived stress, whereas self-efficacy showed a stronger moderating association for the link between perceived stress and loneliness. These differences should not be interpreted as evidence of a verified temporal sequence or causal mechanism; rather, they indicate that the two protective resources were associated with different parts of the proposed pathway in a manner consistent with theoretical expectations. This pattern is in line with the view that different resources may be differentially relevant to stress-related adjustment (Hobfoll, 1989). From an applied perspective, the findings suggest that interventions targeting adolescent depression may benefit from considering both family-level relational resources and individual-level efficacy beliefs. The network analysis further supported this differentiated pattern: self-efficacy showed the highest betweenness centrality among all nodes, identifying it as a key bridge node in the network and providing descriptive support consistent with its potentially important bridging role in the overall pattern of associations (Epskamp et al., 2018).

### 4.4 Limitations and future directions

Despite these findings, several limitations should be noted. First, the cross-sectional design limits causal inference and cannot rule out reverse causality. Future studies should adopt multi-wave longitudinal designs and random-intercept cross-lagged panel models to test the directionality of the chained process more rigorously (Kristensen et al., 2023). Second, the sample was drawn from a single province in northwestern China, which may constrain generalizability given regional differences in educational resources, family structure, and cultural norms. Replication across regions, educational stages, and cultural contexts is therefore needed. Third, all variables were self-reported. Although common method bias was acceptable, responses may still have been affected by social desirability, emotional state, and shared method variance. Future studies could incorporate multi-informant and physiological measures to strengthen validity. Finally, the present analysis relied on observed composite scores and did not explicitly model measurement error. Although the measures showed acceptable internal consistency and prior validation evidence, the precision of the estimated effects may still have been affected by measurement error. Future studies could use latent-variable structural equation modeling to replicate the proposed model while accounting for measurement error.

## 5 Conclusion

The present study showed that different types of academic stress were associated with adolescent depression through a proposed cognitive–interpersonal pathway involving perceived stress and loneliness. Self-imposed stress showed the strongest serial indirect association with depression, possibly reflecting its self-directed and persistent nature. Social stress showed an additional association with loneliness beyond perceived stress, and this loneliness-related indirect association was the largest among all stress types. Parent–child communication and self-efficacy were associated with weaker links at different parts of the proposed pathway: the former was related to a weaker association between self-imposed stress and perceived stress, whereas the latter was related to a weaker association between perceived stress and loneliness. Overall, this study identified differentiated association patterns linking academic stress to depression and proposed an analytical framework integrating stress sources, serial mediators, and node-specific protective resources, thereby providing a useful reference for more targeted interventions for adolescent depression in the Chinese context.

## Data Availability

The raw data supporting the conclusions of this article will be made available by the authors, without undue reservation.
