# Supplementary material for: The serial mediating effects of different types of academic stress on depression among high school students: a two-layer protective mechanism based on conservation of resources theory
Source: Front Psychol. 2026 Jun 30;17:1841587. doi: 10.3389/fpsyg.2026.1841587 (PMC13364565; doi:10.3389/fpsyg.2026.1841587)
Supplement: Supplementary file 1 [file Supplementary_file_1.DOCX]

**Supplementary Materials**

**Overview.** This supplement reports full results of the EBICglasso psychometric network analysis conducted to provide supplementary structural support for the main path-analytic findings. The network was estimated using regularized partial correlations (Extended Bayesian Information Criterion with tuning parameter γ = 0.50). Edge stability was assessed via 500 nonparametric bootstrap iterations; only edges with bootstrapped stability ≥ .50 and |*r*| ≥ .05 were retained. Node centrality stability was evaluated using the case-dropping subset bootstrap procedure (Epskamp et al., 2018).

**Table S1**

*Node Centrality Indices of the Psychometric Network (N* = 967)

| **Node** | **Strength** | **Betweenness** | **Closeness** | **EI** |
| --- | --- | --- | --- | --- |
| Perceived Stress | 1.326 | 0.125 | 0.889 | 1.326 |
| Depression | 1.094 | 0.839 | 0.667 | 0.129 |
| Self-Imposed Stress | 0.902 | 0.536 | 0.800 | 0.472 |
| Loneliness | 0.781 | 0.000 | 0.615 | 0.781 |
| Self-Efficacy | 0.680 | 0.857 | 0.667 | −0.163 |
| Parental Stress | 0.645 | 0.000 | 0.667 | 0.431 |
| Parent-Child Comm. | 0.641 | 0.750 | 0.727 | −0.239 |
| Teacher Stress | 0.565 | 0.161 | 0.727 | 0.451 |
| Social Stress | 0.439 | 0.214 | 0.667 | 0.439 |

Note. *N* = 967. EBICglasso regularized partial correlation network (γ = 0.50). Strength = sum of absolute edge weights connected to a node. Betweenness = number of shortest paths passing through a node. Closeness = inverse of the average shortest path length from a node to all others. EI = Expected Influence (sum of signed edge weights). All indices are raw (unstandardized) values.

**Table S2**

*Edge Weights with 95% Bootstrap Confidence Intervals and Stability Coefficients*

*(N* = 967; 200 bootstrap iterations; sorted by |*r*|, descending)

| **Edge** | **r** | **SE** | **95% CI LL** | **95% CI UL** | **Stability** |
| --- | --- | --- | --- | --- | --- |
| Loneliness — Depression | .429 | .024 | .382 | .470 | 1.00 |
| Parental Stress — Perceived Stress | .346 | .026 | .301 | .395 | 1.00 |
| Self-Imposed Stress — Perceived Stress | .327 | .030 | .266 | .377 | 1.00 |
| Depression — Self-Efficacy | −.258 | .029 | −.310 | −.203 | 1.00 |
| Depression — Parent-Child Comm. | −.224 | .032 | −.274 | −.159 | 1.00 |
| Parent-Child Comm. — Self-Efficacy | .201 | .026 | .158 | .253 | 1.00 |
| Self-Imposed Stress — Teacher Stress | .195 | .030 | .132 | .259 | 1.00 |
| Social Stress — Loneliness | .190 | .031 | .124 | .242 | 1.00 |
| Perceived Stress — Depression | .183 | .026 | .127 | .241 | 1.00 |
| Self-Imposed Stress — Self-Efficacy | −.163 | .027 | −.225 | −.111 | 1.00 |
| Perceived Stress — Loneliness | .162 | .028 | .103 | .216 | 1.00 |
| Teacher Stress — Perceived Stress | .145 | .030 | .087 | .203 | 1.00 |
| Parental Stress — Parent-Child Comm. | −.107 | .029 | −.156 | −.039 | 0.96 |
| Social Stress — Perceived Stress | .106 | .029 | .053 | .162 | 0.97 |
| Parental Stress — Self-Imposed Stress | .098 | .033 | .033 | .148 | 0.94 |
| Parental Stress — Teacher Stress | .093 | .031 | .030 | .156 | 0.91 |
| Teacher Stress — Social Stress | .076 | .030 | .022 | .140 | 0.76 |
| Self-Imposed Stress — Social Stress | .067 | .031 | .005 | .124 | 0.75 |
| Perceived Stress — Self-Efficacy | .057 | .030 | .001 | .121 | 0.63 |
| Teacher Stress — Parent-Child Comm. | −.057 | .032 | −.123 | .000 | 0.52 |
| Self-Imposed Stress — Parent-Child Comm. | −.052 | .028 | −.109 | .000 | 0.54 |

Note. *r* = partial correlation coefficient (EBICglasso). SE = bootstrapped standard error. 95% CI LL/UL = lower/upper limits of 95% bootstrap confidence interval. Stability = proportion of bootstrap samples in which the edge was present (|*r*| ≥ .05). Edges with stability ≥ .50 were retained in the final network (21 edges total; mean stability = .90).

**Table S3**

*Centrality Stability: Correlations Between Full-Sample and Subset Centrality Indices Across Case-Dropping Proportions*

*(N* = 967; 50 bootstrap iterations per subset level)

| **Cases Retained** | **Strength** | **Betweenness** | **Closeness** | **Exp. Influence** |
| --- | --- | --- | --- | --- |
| 90% | .992 | .946 | .886 | .997 |
| 80% | .989 | .941 | .858 | .996 |
| 70% | .981 | .886 | .730 | .993 |
| 60% | .970 | .894 | .660 | .989 |
| 50% | .965 | .889 | .646 | .987 |
| 40% | .955 | .870 | .617 | .983 |
| 30% | .949 | .883 | .550 | .977 |
| **CS-coefficient** | **.30** | **.30** | **.70** | **.30** |

Note. Values represent the average Pearson correlation between full-sample centrality indices and those estimated from bootstrap subsets at each case-retention level. CS-coefficient = largest proportion of cases that can be dropped while maintaining a mean correlation of ≥ .70 with the full-sample centrality order (Epskamp et al., 2018). CS ≥ .25 = minimum acceptable; CS ≥ .50 = ideal. Strength, Betweenness, and Expected Influence (CS = .30) and Closeness (CS = .70) all exceed the minimum threshold.

**Network Description**

The final network comprised 9 nodes and 21 stable edges (density = 0.58). All edges survived EBICglasso regularization, which aggressively penalizes weak or redundant connections, indicating that each retained edge reflects a genuine partial association after accounting for all other variables in the network.

Key findings are as follows:

**Perceived stress as the central hub.** Perceived stress had the highest node strength (1.33) and closeness centrality (0.89) in the network, indicating it is the most strongly and broadly connected node. This structural position is consistent with its role as the primary cognitive mediator in the path model.

**Self-efficacy as the key bridge node.** Self-efficacy had the highest betweenness centrality (0.86), indicating it serves as the most critical bridge between different parts of the network. This finding provides independent structural evidence for its stronger interceptive effect compared to parent–child communication, which had lower betweenness centrality (0.75).

**Social stress direct pathway to loneliness.** Social stress was the only academic stress node with a direct stable edge to loneliness (*r* = .19, stability = 1.00). The other three academic stress nodes (parental, self-imposed, and teacher stress) had no direct stable edge to loneliness after controlling for perceived stress. This pattern independently corroborates the distinctive dual-path mechanism of social stress identified in the path model.

**Strongest edges.** The loneliness–depression edge was the strongest in the network (*r* = .43, 95% CI [.38, .47]), followed by parental stress–perceived stress (*r* = .35) and self-imposed stress–perceived stress (*r* = .33). Both protective resources showed negative edges with depression: self-efficacy (*r* = −.26) and parent–child communication (*r* = −.22).

**Reference**

Epskamp, S., Borsboom, D., & Fried, E. I. (2018). Estimating psychological networks and their accuracy: A tutorial paper. *Behavior Research Methods*, *50*(1), 195–212. https://doi.org/10.3758/s13428-017-0862-1
